# Building strength for the long haul toward liberation: What psychology can contribute to the resilience of communities targeted by state‐sanctioned violence

**DOI:** 10.1002/ajcp.12596

**Published:** 2022-04-06

**Authors:** Kris T. Gebhard, Stephanie Hargrove, Tahani Chaudhry, Syeda Y. Buchwach, Lauren B. Cattaneo

**Affiliations:** ^1^ Department of Psychology George Mason University Fairfax Virginia USA

**Keywords:** community resilience, empowerment, liberation, resilience, state‐sanctioned violence

## Abstract

State‐sanctioned violence (SSV) has resounding effects on entire populations, and marginalized communities have long persisted in the work toward liberation despite continued SSV. This paper aims to bridge the gap between the vast scholarship on resilience and the practical challenge of sustaining and thriving in communities targeted by SSV. We use the theoretical frame of the Transconceptual Model of Empowerment and Resilience (TMER) to articulate the process of resilience and the resources that support it: maintenance, efficacy, skills, knowledge, and community resources. As a practical frame, we ground our application of the model in the experiences of the first two authors in their own communities. Centering examples from the Black Lives Matter movement and the CeCe McDonald Support Committee, we use our theoretical and practical frames to explore the scholarship on resilience relevant to resisting SSV, and we identify mechanisms for supporting community stakeholders' efforts to move toward liberation from SSV. We discuss implications for future research and activism, and we include a toolkit with suggested strategies as an appendix for psychologists, activists, and community stakeholders to consider as they work to facilitate community resilience and build a society free from SSV.

## Highlights

- Adapts findings from resilience literature to inform strategies for community resilience.
- Presents model for community resilience in communities targeted by state‐sanctioned.
- Presents model for resilience in communities targeted by state‐sanctioned violence.
- Black lives matter, Black trans lives matter.

## INTRODUCTION

State‐sanctioned violence (SSV) poses a virulent challenge to the wellbeing of marginalized communities, with impact that radiates far beyond the individuals directly involved. In the spring of 2020, while we were completing this paper, high profile killings by law enforcement in the Black community brought fresh attention to this chronic social issue, prompting large protests across the globe, and breathing renewed energy into calls for criminal justice reform (Danner, 2020; Oppel & Taylor, 2020; Stolberg, 2020). At the time of this writing, these reform efforts have largely been frustrated (e.g., MPR News Staff, 2021).

While the killing of unarmed citizens by police rightly elicits public outrage, it is not the only form of SSV that plagues marginalized communities. SSV is broad term that includes a range of ways government entities enact or respond to violence. In this paper, we define it as government entities' use of violence to control or punish (Patton & Njoku, 2019); those entities' turning a blind eye to violence; and criminal prosecution of those who attempt to defend themselves (Delgado, 2020). SSV against marginalized communities in the United States has been a fixture of the country since its beginnings; as a component of systemic oppression, it extends far beyond discrete incidents (Clarke, 1998; Cooper, 2015; Kendi, 2016). It includes practice and policy related to the criminal legal system at all levels—from law enforcement to the court and the carceral system.

The deeply embedded and systemic nature of SSV means that change does not happen quickly or inexorably; it is likely to happen in fits and starts, requiring long‐term pressure and effort. This long‐term effort must persist in the face of new instances of SSV; communities need to maintain the energy to continue working for change—and living and thriving—within that reality. French et al. (2020) have described a framework of “radical healing” that applies to communities in this situation, asserting that they must “exist in both spaces of resisting oppression and moving toward freedom” (p.11). The importance of maintaining hope and forward motion in that dialectic means that the recognition and facilitation of resilience is an essential ingredient in the work to end SSV.

The centrality of the concept of resilience in this long haul, and in psychologists' potential contributions to it, is clear. For a salient example, in a call to action for counseling psychologists to strengthen their involvement in the Black Lives Matter movement, Hargons et al. (2017) cite the cocreator of #BlackLivesMatter^1^. Alicia Garza (2014) describes the movement as “…an affirmation of Black folks' contributions to this society, our humanity, and our resilience in the face of deadly oppression.” Psychologists aiming to contribute to communities' work in this vein should be able to draw on the vast scholarship about the concept of resilience, including the way it works and how it can be facilitated. However, the focus of much of the existing scholarship falls short of this potential.

The vantage point of the extant resilience literature is often a poor fit for that challenge in several specific ways. First, it often has an individual‐level focus, which misses the systemic nature of a societal issue like SSV. Liberation psychology makes clear the deeply intertwined nature of the structural and psychological aspects of oppression and therefore of liberation (French et al., 2020; Prilleltensky, 2008). When structural oppression is internalized, it has the potential to inhibit efforts to resist; thus liberation from internalized oppression can be both the driver and the result of structural change (e.g., Freire, 2000; Prilleltensky, 2003). To be relevant to SSV, conceptualizations of resilience must capture this complex reality. Second, literature on community resilience often focuses on devastating but isolated events such as a natural disaster or a school shooting. While such events might affect community wellbeing profoundly, responding to an indication and exacerbation of ongoing oppression is a different challenge than putting pieces back together after a lone incident. Finally, much resilience literature assumes that intervention comes from sources outside of communities. While such support can be helpful, this approach misses the opportunity to center the resistance of the communities themselves (Prilleltensky, 2008).

To bridge the gap between the vast scholarship on resilience and the practical challenge of sustaining and thriving in communities targeted by SSV, this paper embraces a qualitative and community‐based perspective on research, as outlined by Brodsky et al. (2016, 2017) and exemplified by Merrick (1999), Fine et al. (2000), and Brodsky et al. (2004). Both community‐based work and qualitative methods are oriented toward questioning dominant narratives and paradigms, prioritizing the expertise arising from lived experience, and identifying the relevance of research for social action (Brodsky et al., 2016). We applied these methods by bringing three sources of information into dialog with each other: the resilience scholarship, a theory of the intertwined processes of empowerment and resilience that was developed from community‐based research (Transconceptual Model of Resilience and Empowerment, TMER; Brodsky & Cattaneo, 2013), and the community‐based perspectives of our first two authors. Both Gebhard and Hargrove were actively engaged in their communities' efforts related to SSV before attending graduate school, and both hoped to use their growing expertise in psychology to support their communities but were frustrated in that effort.

As both participants in the literature review and providers of their community‐based perspectives, our two first authors fall into the “insider‐outsider” dialectic described by Corbin Dwyer and Buckle (2009). Researchers who are members of a group being studied have the deep knowledge that stems from lived experience, and access to social networks and collective wisdom that outsider‐researchers might not have. As researchers, these community members are also on the “outside” looking in. Fine et al. (2000, p. 108) have described inhabiting this in‐between social location as “working the hyphen.” At the hyphen, our first authors occupied both spaces: As psychologists‐in‐training (at the time) they had access to and a growing capacity to understand a scholarly knowledge base, while as community insiders they had lived understanding of the practical issues the scholarship failed to address. This in‐between perspective was not only the reason our team identified the practical challenge that spurred the literature review, it was also crucial to our ability to address it. Centering such perspectives, rather than attempting to remove them from the analysis, is consistent with a community‐based framework and with qualitative and feminist methods, which encourage researcher reflexivity (e.g., Cosgrove & McHugh, 2000). Reflexivity is the range of practices in which researchers consider and potentially report how their own positionality plays a role in the research process. In this way of thinking, “‘research' becomes the collaborative construction of knowledge rather than the discovery of knowledge assumed to already exist” (Probst, 2015, p. 38).

Next, we describe the three sources of knowledge we brought together in this “collaborative construction.” We then articulate the results of this process: a detailed description of mechanisms for supporting community stakeholders' efforts to move toward liberation from SSV. We include a toolkit with suggested strategies for building these resources as an appendix. We conclude with a reflection on the process of bringing these different sources of knowledge into conversation.

## A THEORETICAL FRAME FOR APPLYING THE PROCESS OF RESILIENCE TO SSV

**Figure 1:**
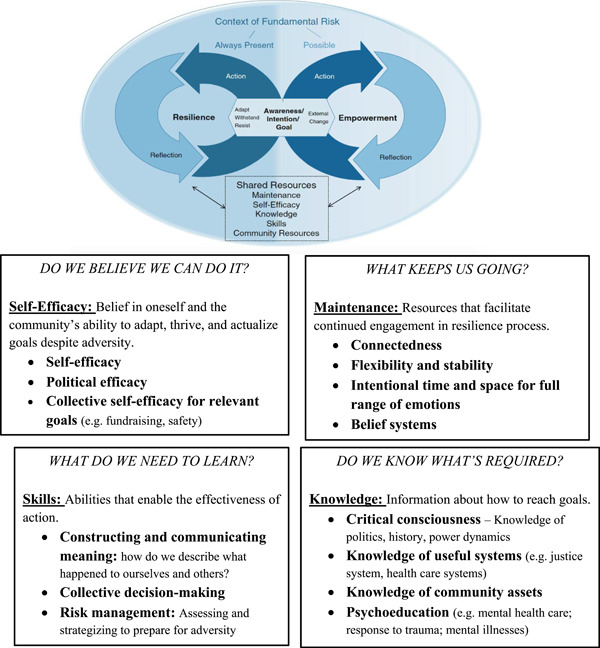
The Transconceptual Model of Resilience and Empowerment (TMER) applied to maintaining community resilience when resisting state‐sanctioned violence [Color figure can be viewed at wileyonlinelibrary.com]

The TMER articulates the intertwined processes of resilience and empowerment, connecting the psychological and social shifts demanded in resisting oppression. As is depicted in Figure 1, both empowerment and resilience are iterative responses to adversity and are fueled by a common set of resources (Brodsky & Cattaneo, 2013). Both empowerment and resilience include an awareness of a problem and the intention to take action, at the group level, at the individual level, or both. Action is followed by reflection about impact, and then a revisiting of intentions. The process of resilience is aimed toward resistance—facilitating strength and wellness in the face of ongoing risk—while the process of empowerment is aimed toward shifts in power. The arduous work of resisting oppression in these ways requires a back and forth between these processes—gaining strength, attempting change, assessing what is needed, caring for each other, and returning to attempting change.

Both empowerment and resilience are relevant processes in the work to end SSV, and because a common set of resources fuels them, strategies to facilitate one also facilitate the other. However, events at the time of the writing of this paper, including both the call for police reform and the backlash to that call, make the particular salience of resilience painfully clear. The ongoing risk to communities, as they work for change, is undeniable. Even if incremental progress is made, the fact that SSV will continue in the meantime means that resilience will be essential. Therefore, while acknowledging the importance of empowerment, we focus here on the untapped potential of the large body of resilience scholarship.

Applied the context of SSV, in the TMER, the anchor of the resilience process is awareness of risk, such that a person or community takes stock of where they are and what is possible. This awareness step, like the rest of the resilience process, is iterative and builds on other steps in the process. Taking action that fails or succeeds, or gathering resources to fuel action, influences one's perspective. Thus, the questions of “where are we?” and “what is possible?” may be revisited regularly. For example, when a specific transgender person is accused of a crime, a community stakeholder assesses risks and possibilities given the particular societal moment, aspects of the case, and resources in the community. If one step to support the accused person fails, there may need to be a regrouping and gathering of different resources to try again, requiring a new awareness.

Resilience action includes the limitless array of steps that might respond to adversity, and reflection is the consideration of what these steps have produced. The TMER resources influence how this process (awareness to action to reflection to a more informed awareness, and so on) evolves. They include self‐efficacy (the belief that one/one's group is capable of accomplishing a goal), skills (the actual capacity to reach a goal), knowledge (possessing the information necessary to take action), community resources (having access to the necessary means for success), and maintenance (the supports needed to remain engaged in the process). How much of any resource is “enough” to fuel action has no static answer; the degree of resources needed depends on the exigency of the moment, in addition to who is doing the acting. These resources might be viewed as questions in evaluating whether and how to act, or in exploring why action has not had the intended effect: “Our group is not functioning well. Might increasing this resource help at this moment?”

As intended by the model's originators (including the last author), the TMER resources are amenable to practical application, as they can be built or degraded over time. The precise nature of these resources manifests differently across situations. Here we apply them to the specific question of how community members continue working toward liberation over the long haul, while shoring up their ability to hope and thrive. The resources provide a tangible point of entry for influencing the resilience process of individuals and communities suffering from SSV (see Figure 1).

This theoretical model provides a framework through which to search the vast resilience literature for findings that could be applicable to the context of resisting SSV. However, as described above, the vantage point of the existing literature is not inclusive of the practical problem we wished to address. Therefore, in the next section, the two first authors will demonstrate the unique perspective of community stakeholders engaged in the continual resistance to SSV, and the need for resilience scholarship to be applied more practically than it has been to date to support their efforts.

## COMMUNITY STAKEHOLDER EXAMPLES ENGAGED IN RESISTANCE TO SSV

Global protests spurred by the killing of George Floyd by police, who suffocated him as he lay prone on the ground and begged for his life in the spring of 2020, show that many people can be affected and moved to action by SSV, that the volume of the protest matters, and that it fluctuates over time. In this context, we define “community stakeholders” as those who understand their wellbeing as intertwined with that of targeted communities, and who are invested in their liberation. While the engagement of many community stakeholders is vital for social change, the role of community stakeholders who also identify as community members is distinct. These internal stakeholders are affected profoundly by the prevalence of SSV in their communities, and they have a challenging and essential role in social change. This role can vary in its formality—from a leadership position in an activist group^2^ to a source of support and connection for a range of community members. Our two first authors provide examples of this internal community stakeholder connection, and the three co‐authors center the experience of the first two. Similarly, this paper is aimed to inform the efforts of all community stakeholders, but centers and prioritizes the experiences of internal community stakeholders.

*Kris T. Gebhard*: I am a genderqueer/transmasculine White individual. Before starting graduate school in psychology, I was an activist organizing community events with trans youth in Minneapolis, MN. In June 2011, CeCe McDonald, an African American transgender woman and community leader, survived a racist and transmisogynist violent hate attack in her neighborhood while walking to the grocery store. After surviving the attack and flagging down police to ask for help to get to the hospital, she was arrested and charged with second degree murder. She was denied proper medical care and held in the male jail in solitary confinement. McDonald reached out to local community leaders, with whom I collaborated to organize the “CeCe Support Committee,” in which I was active in the media subcommittee (Fischer, 2016). I also provided peer mental health support to members of McDonald's queer family and other committee members. Although we accomplished incredible work and engaged in innovative community building, I felt persistently haunted by the belief that we could be more effectively building and maintaining resilience. I often came home after committee meetings feeling drained rather than rejuvenated. Retrospectively, I believe this was due to our focus on the work and negligence of attending more fully to our community health and strength.

Once in graduate school, I connected with trans and queer communities in Washington, DC, and joined with efforts to support GiGi Thomas, an African American trans woman and social worker who faced criminal charges. Believing I now had greater access to resources, in preparation for attending her trial I turned to psychology literature to seek guidance for how to support the resilience of other trans community members attending the court hearing. I was frustrated in that search and felt that existing scholarship could not offer me what I needed to perform the most important component of my community engagement at that moment—supporting the wellbeing and motivation of my community.

*Stephanie Hargrove*: As a Black cisgender woman, seeing people from my community recurrently killed with no regard to their humanity has been horrifying and utterly devastating. Prior to graduate school and throughout my training in clinical psychology, I actively served the Black community in the DC area. I held leadership roles in myriad spaces ranging from my church's social responsibility team to the Greater Washington Urban League's Young Professional Chapter, as Empowerment Academy Chair for youth. I was also deeply embedded in the movement against gender‐based violence, serving as an advocate in domestic violence shelters and as a rape crisis hotline worker. Given my engagement in movements toward social justice, my HBCU upbringing, and my clinical psychology training, I incorrectly assumed I would be well equipped to combat and heal from SSV.

It became clear that I was not effectively navigating the corrosive effects of SSV when Philando Castile was killed by Officer Jeronimo Yanez in July of 2016 and the video of his final moments were released to the public. I watched the video in an airport terminal, waiting to board a flight to a research conference. The devastation and horror resurfaced along with new feelings of demoralization. In that moment I wondered, what was the point of my years of training in social justice advocacy and therapeutic interventions if it did not help me in this moment where I needed healing and hope more than ever? What was the point of attending a research conference if it was not going to attend to this pressing public health issue? I cried, releasing all the emotions that came up in the moment and decided to move forward, boarded the plane and attended the conference with my colleagues. Later we debriefed as a lab and discussed the need for resilience resources to help us endure while fighting for freedom. As researchers, clinicians, advocates, activists, and community members, we were at a loss.

The exemplars just provided represent broad patterns. The prevalence of SSV in the Black and LGBTQIA communities is well‐documented (Delgado, 2020; Human Rights Campaign, 2019), and the fact that any new incident of SSV brings to memory a long history of brutality compounds the impact. SSV instills fear and uncertainty in community members who have witnessed the traumatic circumstances personally, from afar, or even intergenerationally (Bryant‐Davis et al., 2017). Even if allies care deeply about a community, those who share a marginalized identity with the victims of SSV are at higher risk of experiencing negative mental health consequences compared to people who do not share that identity. A strong connection to group values, norms, and traditions leads to the integration of the self and the group into a “group self,” such that violation against one group member impacts the wellbeing of the group as a whole (Volkan, 1999). This phenomenon is especially likely in a community with collectivist values (such as the Black community; Bent‐Goodley, 2005), where SSV can be experienced as cultural or collective trauma (Alexander, 2004; Jackson, 2017). Perhaps for these reasons, in a nationally‐representative sample including over 100,000 Black Americans, researchers found that that police killings of unarmed Black men were associated with significantly poorer mental health among Black men in the state where the killings occurred in the months afterward, compared to no significant mental health impact among the White respondents (Bor et al., 2018). As suggested by our exemplar stories, these data document the sustained assault SSV makes on the wellbeing of marginalized communities, underscoring the need for resilience.

## LITERATURE REVIEW METHOD

As described earlier, the impetus for the paper was the frustrated efforts of our first two authors to use the scholarship to support their own SSV‐targeted communities. We brought the scholarship into conversation with their lived experiences through an iterative conceptual literature review. As a first step, we wished to gain a broad sense of the literature, investigating the impression of our first authors. We read widely in relevant theory (e.g., Luthar et al., 2000; Norris et al., 2008; Walsh, 2003), literature reviews (e.g., Benzies & Mychasiuk, 2009), and empirical work related to experiences of surviving social problems (e.g., Brodsky et al., 2011; Jackson, 2017). As described earlier, we found that much of this resilience literature had a micro‐system focus, utilized interventions coming from outside of communities rather than from within communities, and focused on isolated incidences rather than ongoing oppression. Thus, we also read theory on cultural trauma, historical trauma, and mass trauma (e.g., Alexander, 2004; Onwuachi‐Willig, 2016a). We confirmed that no prior work answered our practical problem.

As a second step, we aimed to develop a list of practically relevant resources. We observed that the resources found in our first step easily fell into the five TMER categories, and so we settled on the TMER as a guiding framework. Team members divided the literature and worked separately to identify resources. As a group, we met regularly to discuss the relevance of each resource gleaned from the literature as well as resources identified by the first two authors from their experiences, and to achieve consensus about which resources were conceptually similar enough that they could be synthesized. We noted no new categories of resources beyond the TMER, but we determined that the original category of community resources (the assets a community possesses that support resilience) could be integrated into the other four categories.

As a third step, we aimed to ensure we had identified literature useful in defining each resource articulated in step two, and in naming strategies for building those resources. We searched APA PsycNet using the name of each resource anywhere in the article and “resilience” as a keyword, focusing on peer‐reviewed literature and community‐based work. In cases when this search returned limited literature, we conducted an additional search using just the resource name, culling the results for literature relevant to resilience as applied in the research. This search resulted in a total of 13,623 peer‐reviewed journal articles and 202 books.

Finally, again bringing this vast literature into conversation with our practical frame, we read abstracts and determined applicability to resilience in the SSV context first indvidually, and then as a team. We considered two questions in winnowing the literature: (1) Did the resource definition work for the practical context of resisting SSV, or could it be elaborated or adapted to do so; and (2) what does empirical evidence suggest about ways that particular strategies could build relevant resources? Through this process we culled the results to 264 articles and books. We then drew on this literature to collaboratively define each resource in the SSV context, and to identify ways to build it on both an individual and group level.

In the next section, we describe each resource we identified through this process, present evidence that the resource supports resilience, and share examples of a way the resource has been enacted in Black Lives Matter activism and/or the CeCe Support Committee. These examples highlight the fact that many resources already exist in communities working in this context—in these cases naming them offers the opportunity to build on what is already there. We organize suggested strategies for building resilience resources into a tool kit, which we include as an appendix.

## RESILIENCE RESOURCES IN THE CONTEXT OF SSV

### Maintenance in the context of state‐sanctioned violence

Maintenance resources keep one engaged in the process of resilience (Brodsky & Cattaneo, 2013). They fuel individuals and groups to persist in taking action, evaluating the success of their actions, and reassessing what is possible; they also bolster the strength and effectiveness of other resources. In the context of SSV, key maintenance resources are *connectedness, flexibility and stability*, and *intentional time and space for the full range of emotions*.

#### Connectedness

The resilience literature highlights the power of constructs related to connectedness, such as sense of community (Landau & Saul, 2004; Pfefferbaum et al., 2008), social capital (Aldrich, 2017; Madsen & O'Mullan, 2016), “sense of togetherness and community” (Kulig et al., 2008, p. 94), and belonging (DiFulvio, 2011). These resources help the community to address divisions, take action, and problem‐solve. Literature on stigmatized social identities shows that those with strong connection to the identity‐based community have increased resilience to stigma (e.g., Bogart et al., 2018; Silverman et al., 2017). Brodsky et al. (2012) highlight how membership in a community organization buttressed both the individual and family resilience of the Afghan women in their study; this connectedness supported women in their work resisting SSV.

##### Community stakeholder example

Several members of the CeCe Support Committee had activist experience within the prison abolitionist movement and shared lessons learned and formative thinking from this movement, as well as relationship connections; members reported feeling connected to the movement thanks to shared values, goals, and personal ties. The CeCe Support Committee also connected via social media, direct communication, and philosophically with the larger national and international population of trans survivors of interpersonal violence (Fischer, 2016). This connectedness helped the CeCe Support Committee link their immediate cause with larger purposes, engendering both increased motivation and practical support to take action.

#### Flexibility and stability

After a traumatic experience, stability provides a secure base from which to move forward. Flexibility allows for possibilities in that movement forward, supporting creativity in finding strategies that work. Flexibility and stability are thus intertwined in their enabling of more effective action. Stability can be provided through continuing rituals and practices, maintaining meeting location and/or meeting times, ensuring financial stability, and/or stability in relationships. Community groups that allow for flexible responsiveness to challenges while maintaining a basic level of stability facilitate individual flexibility and stability among their members.

The resilience literature highlights the benefits of flexibility and stability when facing challenging situations. Family resilience literature describes the need for families to flexibly “reallocate roles and adapt to changed conditions,” while simultaneously working to “restore order, safety, and stability” to mitigate the disorienting nature of traumatic events (Walsh, 2007, p. 213). In experimental work with individuals, people who reported a high level of trait resilience demonstrated more emotional flexibility (Waugh et al., 2011), and expressive flexibility moderated the relationship between life stressors and adjustment (Westphal et al., 2010). In other words, being able to respond to varying stimuli and yet stably regulate one's emotions supports one's ability to navigate challenging situations.

##### Community stakeholder example

A source of stability for the CeCe Support committee was maintaining regularly scheduled meetings (often weekly) throughout the year, which provided a stable place to process events and continue longer‐term planning. When CeCe was bonded out of jail, the committee needed to be flexible in this arrangement, moving the meeting to where CeCe was staying so she could participate. By nature of being decentralized, the Black Lives Matter movement is inherently flexible and adaptive to local needs and organizing. The stability of BLM comes from consistent messaging connected with on‐the‐ground actions. There is a strong association between the hashtag and the focus of the movement, which functions to connect otherwise disconnected events, protests, and calls for action. When the hashtag or associated slogans (e.g., hands up don't shoot) are invoked, it is immediately clear what the injustice is that is being highlighted, allowing the movement to continue in a cohesive way.

#### Intentional time and space for the full range of emotions

In the wake of SSV, both individuals and groups are faced with the task of processing emotion. Creating space for community members to feel and share negative emotions such as anger, sadness, and grief is crucial to mental health and can allow those emotions to energize action rather than paralyze and demoralize. On the other end of the continuum, regularly experiencing positive emotions such as gratitude, enjoyment, humor, and joy in community with others can contribute to other resilience resources (e.g., connectedness) and can help shore up strength for managing the grueling long‐term nature of social change. The intentionality of the time and space for the full range of emotions refers to thoughtful management of these complex reactions.

There is significant evidence highlighting the importance of processing a variety of emotions in the aftermath of trauma as a component of healing. Multiple scholars have linked positive emotions with resilience in the context of a broad range of adverse experiences (Arewasikporn et al., 2018; Bonanno, 2004; Fredrickson et al., 2003), but Mancini and Bonanno (2009) also point out that emotional disassociation or repressive coping can be maladaptive over the long‐term. In fact, Bonanno et al. (2011) found that both the ability to focus on processing a trauma and the ability to look toward the future were independently related to better adjustment. In sum, the literature supports the need to experience the full range of emotions.

##### Community stakeholder example

Intentional time and space for the full range of emotions includes space for processing trauma as well as making room for joy. Emotional Emancipation Circles were created to foster the liberation of Black people through raising critical consciousness as well as creating safe spaces for people in the Black community to express their feelings and come together to support each other among the environment of perpetual racism, oppression, and community violence (Grills, Aird, & Rowe, 2016). These circles validate the need to intentionally process emotions within a critical context and historically relevant framework.

This experience is both a reprieve from the onslaught and a way to lighten the load, so that one can continue. On the other end of the spectrum, when community members gathered to support CeCe McDonald for an organized protest or press conference outside the courthouse, they sometimes played music and danced. Expressing joyful resistance and identity celebration before shifting into the more emotionally challenging spaces of a press conference or court hearing gave members the energy to strategically withstand silencing. Intentionally creating space for expressing the full range of emotions enables community stakeholders to more resiliently engage with all aspects of their organizing.

### Self‐efficacy in the context of state‐sanctioned violence

Self‐efficacy, the belief that one is capable of accomplishing specific goals, both motivates action (Locke & Latham, 2002) and makes action more effective (Bandura, 1982; Norris et al., 2008). It is a well‐established construct that can apply both to individuals and groups, where *perceived collective efficacy* is a group's “shared belief in their collective power to produce desired results” (Bandura, 2000).

Several studies support a link between self‐efficacy and resilience. With respect to political goals (*political efficacy*), studies on individuals experiencing adversity from race‐related social conflict or unfair treatment from the government have shown that those with a higher sense of efficacy are more likely to be civically active (Hope & Jagers, 2014; Leath & Chavous, 2017; Zeng et al., 2018). Researchers considering efficacy at the group level explored the aftermath of an earthquake in Nepal, and found that an individual's belief that the country collectively was strong and capable predicted posttraumatic stress and posttraumatic growth (Muldoon et al., 2017). In sum, research supports the idea that at the individual and group levels, a belief that oneself or one's group is capable of attaining set goals can promote both engagement in resilience actions and the success of those actions (Bandura, 2000).

#### Community stakeholder examples

In the context of SSV in which state violence is consistent and unyielding, observing small successes can be crucial to maintain motivation for continuing to work toward the larger vision of systemic change. As police killings of unarmed Black people have persisted and received news coverage during the writing of this paper, there have been some legislative changes and reform efforts in specific police departments, but not (at this point) the large‐scale changes activists want. Organizations can track and celebrate these changes, even while continuing to fight for more profound transformation (Subramanian & Arzy, 2021). Centering the power of groups in making change, many activists have celebrated small accomplishments within the community. For example, an activist observed the care expressed at a protest, writing, “people…set up a security team to keep gatherers safe, there was food and coffee and handwarmers. There were medics and a music truck, and EVERYBODY was wearing a mask (almost none of the cops were)” (S. June, personal communication, December 31, 2020). These successes (the establishment of a security team, provision of sustenance and health care) cumulatively strengthen their hope and belief that the community can build a better world. For the CeCe Support Committee, raising enough funds and community resources to bond CeCe out of jail was a significant achievement for CeCe and the group to celebrate. Her presence at meetings from that point on bolstered motivation while the trial continued.

### Skills in the context of state‐sanctioned violence

Within the TMER, another category of resources is *skills*, or the specific abilities that make action effective. In addition to utilizing skills in their own resilience processes, community members may offer pre‐existing skills to a group, may be recruited to a group effort on the basis of those skills, or may learn skills as a component of their participation. Key skills in the context of SSV include *constructing and communicating meaning, risk management*, and *collective decision‐making*.

#### Constructing and communicating meaning

When a community experiences adversity, meaning‐making is the creation of “communal narratives that give the shared experience meaning and purpose” (Norris et al., 2008, p. 140). These narratives answer questions about “a) the nature of the pain; b) the nature of the victim; [and c) the] relation of the trauma victim to the wider audience” (Alexander, 2004, p. 13). In the case of SSV, these narratives often refute mainstream stories that rationalize and perpetuate violence (Onwuachi‐Willig, 2016b). They articulate the systemic forces at play (Smelser, 2004) and call for action. There is a need to construct meaning within the inner circles of those directly impacted, communicate meaning through social media connecting community members more broadly, and frame messages in mainstream media that inform and influence the general population.

Trauma scholarship clearly conveys that making sense of what has happened while tying the experience to a broader understanding of the self and the world is a key step in moving forward constructively (Holliday et al., 2018). Research highlights the need for attributing the causes of traumatic events and for a continual reframing of stressors “into constructs that motivate rather than devastate” (Brodsky, 1999, p. 157; Brodsky et al., 2011; Welsh & Brodsky, 2010) so that the stressor is “comprehensible, meaningful, and manageable” (Walsh, 2003, p. 7). Collective memory encompasses the material, social, and experiential ways in which groups process past traumatic experiences, make meaning, and shore up group identity. Collective memory of these atrocities, including responses to them and triumphs despite them, can be an important resource to fuel meaning‐making and stoke pride in the group's shared identity (Villenas & Deyhle, 1999). Scholars on collective trauma highlight Max Weber's (1968) description of “carrier groups” (as cited in Alexander, 2004) as leaders who publicly present narratives making meaning of events. These carrier groups can message alternative narratives to the status quo, communicating the “collective pain” of the marginalized population, demanding change, and presenting solutions to prevent future pain (Onwuachi‐Willig, 2016b, pp. 339).

##### Community stakeholder example

Black Lives Matter has a website that shares videos from activists on the frontlines, news written from the perspective of the activists about incidences of SSV in the past and present, and information about other cultural programs relevant to the Black community (Black Lives Matter, 2021). Their content is written by Black activists to inform the Black community, ensuring that the narrative is not reshaped by groups that do not have the same shared history or investment in the community and its members. The CeCe Support Committee's blog website served as an important space to post announcements and archive press releases and media related to CeCe's case that reflected the messaging that the Committee had carefully constructed. Spaces that store the community's collective memory of events have the potential to support resilience through generations.

#### Collective decision‐making

When people have gathered in response to a traumatic event, they will likely need to decide on strategies both to support those most affected by the event and to effect change. Collective decision‐making refers to the collaborative process used to reach such decisions.

The value of skillful collective decision‐making is supported by the resilience literature. In a unique study, Polletta and Hoban (2016) traced the use of consensus decision‐making by activists through the 1940s, 1960s, and 2010s, and interviewed 30 activists from a diversity of organizing contexts whose organizations employed consensus‐based decision‐making models. Participants viewed consensus decision‐making as “a place to work through inequalities that are informal, unacknowledged, and pervasive” (p. 297), supporting the long‐term health of the organizations. Resilience literature shows that agency and participation in making important decisions, and even the act of learning decision‐making skills, contribute to individual, family, and community resilience (e.g., McCrea et al., 2016; Norris & Stevens, 2007; Oliver et al., 2006; Stewart et al., 2004). In sum, the process and outcome of this skill contribute to the resilience of a group and its members.

##### Community stakeholder example

Through its decentralized model, Black Lives Matter movement has remained committed to on‐the‐ground collective decision‐making in which individual city chapters decide their focus and organizing goals (Shaw, 2020). This delegative process intentionally seeks to distribute power more equitably, as opposed to traditional hierarchical models which perpetuate inequality.

#### Risk management

After an event that highlights a community's vulnerability to SSV, community members may be painfully aware of the possibility that future violence could affect them or their loved ones. Risk management is the identification of risk factors that can be changed, and the development of a plan to decrease the likelihood of harm. For those vulnerable to being targeted by violence, effective risk management plans must take into account the full picture of their lives (Cattaneo & Goodman, 2009; Hamby, 2014). In the context of SSV, Black Americans face risk daily, as police killings have occurred in seemingly mundane situations such as being stopped for a traffic violation, or in the case of Breonna Taylor, sleeping in one's bed (Gupta & Hauser, 2020). CeCe McDonald's ordeal occurred while she was walking to the grocery store (Rubin Erdely, 2014). All communities who are targeted by SSV risk the mental health impact of encountering stories or searing imagery of SSV against their community. Finally, social action itself presents risks, whether working to protect victims or advocating for change. Risk management involves identifying such risks and creating plans to manage them in context.

Risk management skills and related safety plans are highlighted in literature regarding the treatment of trauma survivors. As Walsh (2007) notes, learning from a trauma experience can help survivors proactively prepare for and address future threats. Individual and family resilience literature highlights the need for survivors to learn to differentiate between trauma reminders and true risks. Trauma treatment literature also supports risk management in cases where the trauma is ongoing, such as in the case of children who witness domestic violence. The scholarship highlights the importance of risk management not only for decreasing physical risk, but also for shoring up emotional wellbeing, as trauma “shatters a sense of safety because of overwhelming, uncontainable fear” (Classen & Clark, 2017, pp. 523). Classen and Clark (2017) discuss the need for individuals to feel a sense of agency in defining what is safe for them, while naming and addressing anything that makes them feel unsafe.

Community resilience literature focusing on natural disasters highlights the importance of having communication systems in place to warn people about danger and direct people to help (Cutter et al., 2008; Gissing et al., 2010; Thomalla & Larsen, 2010). Better communication before and after events has been associated with a stronger perception of resilience in the community (Spialek & Houston, 2018). In her review of resilience and communication in unpredictable circumstances such as terrorism and natural disasters, Longstaff (2005) emphasizes the need for trusted sources of information even during stable periods because during periods of instability, individuals do not have the time to locate and check new sources. In sum, scholarship suggests that to effectively manage risk and shore up resilience, communities must have access to accurate information and must have the opportunity to develop plans.

##### Community stakeholder example

In the context of SSV, many cases may require expertise to identify risks. In the CeCe Support Committee, lawyers helped the group understand the importance of not sharing details of the case until they had been revealed in court, and they worked with the group to identify messaging that would minimize risk to the case. Black Lives Matter groups regularly distribute to protesters information about how to respond to tear gas as well as information on legal rights (e.g., Elias, 2020). Community members trained in providing medical care are now routinely present for protests. Thinking through how to prepare for and mitigate possible risks enables community members to feel simultaneously more in control of their safety, and more aware of what is outside of their control, allowing for careful decision‐making while working toward change.

### Knowledge in the context of state‐sanctioned violence

The TMER category of knowledge includes all of the information necessary to support resilience in a particular context (Brodsky & Cattaneo, 2013). In the context of SSV, several specific areas of knowledge are likely to be useful and are detailed in this section: “Knowledge of systems,” “Knowledge of community assets,” “Psychoeducation,” and “Critical consciousness.”

#### Knowledge of systems

In the context of SSV, individuals and groups need to understand how to navigate systems when drawn into them, or when they are a necessary mechanism for action. Such systems might include the criminal justice system, the media, and government offices regulating the use of public spaces.

While we did not find knowledge named as such in the resilience literature, Vesely et al. (2017) describe the importance of “navigational capital” in the resilience of immigrant families. In their conceptual model of contextual factors influencing resilience for this population, the understanding of how to access and make use of systems is a powerful facilitator, while a lack of this understanding creates a formidable obstacle.

##### Community stakeholder example

The CeCe Support Committee's efforts to connect to larger organizations exemplify how larger or more established organizations can increase smaller groups' access to relevant expertise (Skertich et al., 2013). The CeCe Support media committee received a media training from staff at the National Coalition for Transgender Equality. The training increased the group's knowledge of how to effectively frame stories in ways that could benefit CeCe's case when generating mainstream media.

#### Knowledge of community assets

To achieve their goals, community members must know what assets they have at their disposal. Community assets can include finances to fund events and support community members, space to host meetings and other events, community members who are skilled in particular areas, and community members who either have influence in particular circles or have relationships with people in positions of power (also referred to as social capital; Aldrich & Meyer, 2015). In order for community members or organized groups to draw on these assets, they must know that they exist. We did not find mention of this resource in the resilience literature.

##### Community stakeholder example

Many local Black Lives Matter chapters have a community‐based resources list on their website to easily share information on local legal support sources, local organizations that are working for a similar cause, and local mental health resources (e.g., https://phillywerise.com/blog/resources/, 2021). Sharing this knowledge of community assets enables people to efficiently find resources and services with professionals who align with their values.

#### Psychoeducation

Trauma‐focused psychoeducation helps an individual or group understand ways that traumatic experiences can affect one's functioning and imparts strategies for managing traumatic stress and healing from traumatic experiences (Whitworth, 2016). In the context of SSV, this information could include the understanding that one can be deeply affected by the targeting of one's community, even if one does not personally know people who were physically harmed.

Psychoeducation is a crucial component in evidence‐based treatments for posttraumatic stress disorder and is similarly important for individuals dealing with an ongoing traumatic stress response (Diamond et al., 2013). In the SSV context, psychoeducation can focus on validating community members' emotional response to the trauma (Diamond et al., 2013) and can also inform coping strategies. Indeed, numerous studies have utilized psychoeducational interventions to enhance resilience in trauma survivors (Besani et al., 2018; Myers‐Coffman et al., 2020), and trauma‐informed cognitive‐behavioral therapy (TF‐CBT), which explicitly includes psychoeducation as a core component, has been successfully used with youth and families experiencing ongoing traumas (Murray et al., 2013).

##### Community stakeholder example

In the aftermath of the murder of George Floyd, community stakeholders worked quickly to publicize information about the impact of SSV on the Black community (and much more; Academics for Black Lives, 2020; Jernigan et al., 2015). More broadly, an online toolkit created by Community Healing Network, Emotional Emancipation Circles, and the Community Healing Network et al., (2016) has useful tables on “Trauma Reaction” at the individual, community, and societal levels (pp. 19–20) and a list of “Common Reactions to Stress and Trauma—Things to Look Out For” (p. 8). These tables provide helpful psychoeducation on the impact of trauma.

#### Critical consciousness

Freire (2000) defined critical consciousness as "learning to perceive social, political, and economic contradictions and to take action against the oppressive elements of reality" (p.35). It has been divided into three components: critical reflection, critical motivation, and critical action (Diemer et al., 2016). While its action‐oriented components are often viewed as particularly relevant to the process of empowerment (Diemer, et al., 2015), the first two components might build resilience in communities in the aftermath of SSV—understanding a single incident in sociopolitical context, and fueling determination.

Scholarship has identified critical consciousness as a resource for coping and healing from trauma and adversity for marginalized communities (Bartholomew et al., 2018; Noyola et al., 2020). French and colleagues' (2020) psychological framework for radical healing from racial trauma in communities of color defines radical healing as “both acknowledgment of and active resistance from oppression, as well as a vision of possibilities for freedom and wellness” (p. 24). Critical consciousness is identified as the first step toward radical healing in this framework because awareness of the current oppressive systems can foster hope and lead communities of color to envision a better future. Building the resource of critical consciousness supports resilience in the face of ongoing oppression.

##### Community stakeholder example

The Black Lives Matter movement has developed a framework called “healing in action” (Bartholomew et al., 2018), which encourages individuals and groups to engage in practices that build and maintain critical consciousness before, during, and after an action. In the first step, when group members are preparing for an action, they are encouraged to engage in centering and grounding exercises—such as breathing exercises, chanting, and check‐ins—and visioning exercises, in which group members reaffirm the group vision for Black liberation. The second step, during the action, includes group members' dialog and the pursuit of a deeper connection with the community, facilitating critical reflection and awareness of ways trauma can impact their work. The third step after the action is completed allows for individuals and groups to collectively process, again facilitating critical reflection. The framework integrates exercises that enable group members to engage in critical reflection throughout the critical action.

## DISCUSSION

### Community stakeholder reflection

Throughout our discussion of resources, we highlighted examples from the CeCe Support Committee and/or Black Lives Matter contexts. Both Gebhard and Hargrove contributed many of these examples informed by our experiences organizing in these contexts; our insights also informed the strategy ideas for building resources highlighted in the Toolkit (Appendix A). The process of mining the literature, discussing with each other and the rest of the team and then articulating resilience resources and strategies to build them was revealing and affirming. For both Gebhard and Hargrove, this process deepened our insight into our past experiences, and has enabled us to imagine bringing expertise to organizing spaces as both psychologists and community members with organizing experience. We appreciated finding language to articulate some of the resilience resources that we were most grateful to have built during our organizing experiences, as well as resources we would like to have built more. As organizers who came to psychology to help the field better resource our communities, we also found it affirming to bring our underrepresented perspectives into dialog with the literature to adapt resilient resources to the context of resisting SSV.

### Implications for the use of the TMER

We found the TMER to be a helpful organizing framework for bringing a vast scholarship into conversation with the community‐based experiences of our authors. The resource categories were generally helpful in considering the practical relevance of scholarship, and we expect that the TMER might be similarly useful in approaching other topics. A question for these future applications is whether the category of “community resources” might be most efficiently integrated into the other resources as it was here. In future work applying the TMER to SSV, the nuances of the intertwined nature of resilience and empowerment in the context of SSV might be explored, potentially yielding additional insight into the ways psychology can support social change. Further, although we found individual, family, and/or community resilience research related to most resources, we noted that research related to knowledge of systems and knowledge of community assets was especially scarce, and more research is needed in these areas.

## CONCLUSION

To sustain the long haul toward liberation, community stakeholders must build resilience for themselves and their communities while they engage in healing and active resistance to state‐sanctioned violence. This paper used the TMER as a framework to mine the resilience scholarship, distilling the resources applicable for community stakeholders impacted by SSV. Through the frame of reference of two of our authors, we highlighted two touchstone community examples of groups who fostered and are fostering resilience and pursuing liberation. Black Lives Matter activism and the CeCe McDonald Support Committee are examples of resistance groups from two specific communities affected by SSV representing national and local movements, respectively. In using these very different examples, we aimed to show the broad relevance of resilience resources to community stakeholders and groups working in a variety of ways to end SSV.

SSV is a broader category than the one we described in this review, in which we focused on criminal justice. It could be considered to include environmental injustice (e.g., pollution levels being allowed to persist beyond legal levels), economic marginalization, evictions, and displacement. The suggestions presented here may be applicable for communities experiencing these forms of violent assault as well, and we hope others might explore that possibility.

In this paper, we highlight a paradigm for communities contending with their circumstances by working to build internal sustainability rather than needing to trust the state to provide safety, healing, or justice. As is clear in the examples presented in each resource category, our assumption is that communities are already fostering, and have the capability to intentionally actualize, these resilience resources. Allies can support this study; our focus on resourcing internal community stakeholders is not meant to place the burden on communities who experience SSV, but rather to center their expertise and power to resist.

Communities must sustain themselves while they pursue the long fight for justice with the ultimate objective of building a society where people can live free from violence and oppression.

## CONFLICTS OF INTEREST

The authors declare no conflicts of interest.

## Footnotes

^1^
The cocreators of the #Blacklivesmatter hashtag are Alicia Garza, Patrisse Cullors, and Opal Tometi (“Herstory,” n.d., retrieved from https://blacklivesmatter.com/herstory/).

^2^
In this paper, we use the term group to broadly describe any gathering of people who have come together in response to an incident of SSV and/or in support of ongoing organizing regarding SSV. They may gather formally or informally, in person or online, and they may have more or less explicit membership expectations.

## APPENDIX A TOOL KIT: STRATEGIES FOR BUILDING RESILIENCE RESOURCES IN THE CONTEXT OF SSV

This tool kit is a companion to *Building Strength for the Long Haul toward Liberation: What Psychology Can Contribute to the Resilience of Communities Targeted by State‐Sanctioned Violence*. It lists specific strategies that might build each resilience resource described in the paper. Strategies are gleaned from resilience literature and the activist experience of the authors—their utility at any given time depends on context. The strategies are meant to be useful both for individuals working within communities and for groups who have come together in some form to address SSV.

- Resilience Resource Category: Maintenance
  o In order to build Connectedness, stakeholders might…
    ▪ *Share stories in community*.
      - Process experiences related to SSV with others in person or via online platforms, either informally or in a structured format (Crowley, 2014; Jackson, 2017).
      - Build a sense of connectedness by linking the present to the past through personal conversation, art, literature, and forums for elders to share their experiences with similar challenges in the past (Landau, Mittal, & Wieling, 2008; Seaburn, Landau, & Horowitz, 1995).
    ▪ *Connect at community events*.
      - Hold community events, which can allow for communal grieving or celebration or creativity, resilience, and thriving. These events can take the form of protests or a space for expressive writing, sharing art or sharing stories by elders in the community (Jackson, 2017).
  o In order to build flexibility and stability, stakeholders might…
    ▪ *Maintain routine*.
      - Attend to one's normal routine during times of crisis to help ground oneself and reduce the disorienting impact of crisis (Bonanno et al., 2011).
      - Identify one or more regular group practices, like a guided meditation, reading, or check‐in, and practice it at every gathering.
    ▪ *Identify or build anchoring beliefs*.
      - Identify and articulate values and grounding belief systems (see Acceptance and Commitment Therapy (ACT): https://workingwithact.com/2011/11/01/finding-true-north-how-to-clarify-values-part-2/)
      - Refer to values and belief systems when making choices about how to engage in social action or self‐care.
      - Keep grounding values at the forefront of your work by making them concrete and accessible, posting them online, or creating slogans that reflect them.
  o In order to build the Individual Time and Space for the Full Range of Emotions, stakeholders might…
    ▪ *Openly acknowledge and discuss*.
      - Discuss the reality that it is normal and healthy to experience the full range of emotions, both the positive and the negative, and they are necessary as fuel for connection and resilience.
      - Share common responses to trauma and the principles of trauma‐informed care (Community Healing Network et al., 2016).
      - Create structured group activities to help community members regulate and express their feelings, and come together to support each other among the environment of oppression and community violence.
    ▪ *Cope ahead*.
      - Anticipate how one may emotionally react to certain events and identify coping strategies in advance (Linehan, 2015).
      - Discuss the likelihood of particular responses, and plan for how to channel them.
    ▪ *Make and share art*.
      - Produce, share, and discuss art in order to process and communicate emotions. Community events such as poetry open mics, spoken word festivals and block parties (physical forms of art such as krumping) can allow for emotional catharsis, particularly when that art speaks to the issues the group is activating around.

- Resilience Resource Category: Efficacy
  o In order to build Perceived Collective Efficacy, stakeholders might…
    ▪ *Celebrate mastery experiences*.
      - Celebrate small successes in order to increase the sense that the community can reach its goals (Bandura, 1997).
      - Acknowledge and embrace the fact that existing can be a form of mastery: many LGBTQ groups incorporate the idea that “existence is resistance,” demonstrating the powerful impact of each member's participation in the group.
    ▪ *Identify models*.
      - Highlight the success of other similar individuals and groups (“models”).
      - Seek out examples of success by others in your identity group (Because of Them, We Can, https://www.becauseofthemwecan.com), and other marginalized groups that have successfully achieved social change.
      - Be mindful of one's own capacity to serve as a model, sharing one's struggles, techniques for healing, and achievements.

- Resilience Resource Category: Skills
  o In order to build Constructing and Communicating Meaning, stakeholders might
    ▪ *Construct narratives*.
      - Develop narratives that describe the community's experience, and then frame and communicate those narratives broadly (Saltzman, 2016; Saltzman, Pynoos, Lester, Layne, & Beardslee, 2013).
      - Encourage expressive writing, a simple but powerful strategy to help community members make sense of their trauma for themselves. This involves writing uninterrupted for 15 min a day, either about any topic that comes to them, without stopping to edit or analyze, or about a single issue that is important to them where they would like to explore associated thoughts and emotions (Crowley, 2014; Pennebaker, 1997).
      - Create shared meaning with family members (Saltzman, Pynoos, Lester, Layne, & Beardslee, 2013; Saltzman, 2016).
    ▪ *Create a safe space to share and preserve collective narratives*.
      - Create a space that can be figurative, by cultivating an atmosphere of openness to share memories, or literal, by holding gatherings or creating networks designated for disseminating and reinforcing storytelling and collective memory (Corntassel, 2009; Millar, 2006).
      - Create a virtual space to preserve collective memory; this can also support the construction and perpetuation of meaning with easy access for those who are geographically distant.
    ▪ *Translate narratives for social and mainstream media*.
      - Promote messaging through social media in order to gather and sustain collective memory.
      - Encourage posting and messaging as part of an effort to generate mainstream media framed by community perspectives rather than dominant status‐quo discourse.
      - Ask community members with media experience to distill and translate communities' collective narratives into external‐facing messages for social media and/or mainstream media.
      - Agree in advance on basic principles and messaging to uphold when interfacing with social media and mainstream media; this may require consulting with lawyers to align messaging with legal strategy.
  o In order to build Collective Decision‐Making, stakeholders might…
    ▪ *Intentionally choose a decision‐making model*.
      - Choose a model to use for making decisions.
      - Ensure that all group members are educated about the decision‐making process being used and that new members are quickly informed of how to engage.
      - Consider a consensus‐based model, a decision‐making process that brings together diverse voices to discuss and revise proposals, bring forth disagreements, and design a plan of action that values everyone's voices (Hartnett, n.d.; Seeds for Change, 2020).
  o In order to build Risk Management, stakeholders might…
    ▪ *Define safety*.
      - Articulate what safety means in a particular time and context. The concept of safety can vary; for some, having space in which they can get away from reminders of SSV, such as violent imagery or video, might represent safety, whereas for others, anonymity might represent safety.
      - Regularly check in on community members' concept of safety as it may change over time, as the local or national context around SSV shifts.
    ▪ *Identify risks and determine what can be changed*.
      - Engage in scenario planning: Brainstorm scenarios in which your safety is compromised, and then identify the factors in these scenarios that can be changed. As part of this process, individuals and groups can decide how much risk they are willing to tolerate and can revisit this decision on an ongoing basis.
      - Seek help from experts to identify risks, such as understanding potential legal or medical hazards. With respect to psychological risk, when SSV is garnering significant media attention, people can make conscious choices about their exposure.
    ▪ *Agree on a plan to minimize risk*.
      - Plan strategies ahead of time to minimize risk in particular situations and revisit these plans as the context changes (e.g., Elias, 2020).
      - Identify trusted sources of information and agree upon systems of disseminating information in order to keep up with evolving risks.

- Resilience Resource Category: Knowledge
  o In order to build Knowledge of Useful Systems, stakeholders might…
    ▪ *Connect with expertise*.
      - Tap into individual networks and local community organizations that have expertise.
      - Ask individuals with specialized expertise (e.g., activists, lawyers, psychologists, and strategic communications professionals) to lend their knowledge to community organizations or help develop accessible brochures or factsheets to disseminate online.
      - For smaller organizations, connect with larger or more established organizations to increase access to relevant expertise (Skertich et al., 2013).
  o In order to build Knowledge of Community Assets, stakeholders might…
    ▪ *Create a resource hub*.
      - Create resource lists of community assets and community members with specialized expertise to be made accessible to the broader community.
      - Designate a position of maintaining knowledge of community assets and serve as a point person to link community members to assets they are in need of.
  o In order to build Psychoeducation, stakeholders might…
    ▪ *Increase access to trauma‐informed knowledge*.
      - Build knowledge of the impact of trauma through reading and consultation (Community Healing Network et al., 2016; Academics for Black Lives, 2020; Jernigan et al., 2015; https://theresiliencetoolkit.co/)
      - Share information through personal networks and online presence (Whitworth, 2016).
    ▪ *Psychoeducation groups*.
      - Implement structured, nonclinical, psychoeducation groups as outlined by Miller and Wang (2018). The model consists of three sessions of group psychoeducation.
        o The first session includes a discussion of social identity, power and privilege, and the ways that social disasters can cause trauma; this session also allows group members to share their own reactions.
        o The second session entails adapting trauma management strategies to a context in which the threat is ongoing, including self‐calming, mindfulness, and self‐care strategies.
        o The final session reviews and reiterates what group members have learned, giving them a space to practice strategies and plan for the future, including identifying barriers to their healing.
        o Such a group can be a time‐effective strategy to introduce psychoeducation to community members in a non‐therapy context.
  o In order to build Critical Consciousness, stakeholders might….
    ▪ *Develop consciousness in connection*.
      - Engage with critically engaging reading materials, educational podcasts, and documentaries (e.g., Justice in June, https://justiceinjune.org/).
      - Journal—this can help promote deeper thinking and retention of learning.
      - Connect with other community members through facilitated group formats to enhance your critical consciousness through dialogue, engagement, and learning (Bartholomew et al., 2018; Diemer et al., 2016).
